# Personalised Psychological Care in Hospitals: An Organisational Model of Integrated, Patient- and Staff-Centred Services (2019–2024)

**DOI:** 10.3390/jpm16010030

**Published:** 2026-01-05

**Authors:** Daniela Pia Rosaria Chieffo, Valentina Massaroni, Valentina Delle Donne, Letizia Lafuenti, Laura Monti, Valentina Arcangeli, Federica Moriconi, Daniele Ferrarese, Roberta Galluzzi, Eugenio Maria Mercuri, Gabriele Sani, Giampaolo Tortora, Antonio Gasbarrini

**Affiliations:** 1 Clinical Psychology Unit, Fondazione Policlinico Universitario Agostino Gemelli IRCCS, Largo Francesco Vito 1, 00168 Rome, Italy; 2 Complex Operational Unit of Child Neuropsychiatry, Fondazione Policlinico Universitario Agostino Gemelli IRCCS, 00168 Rome, Italy; 3 Department Women Children and Public Health, Università Cattolica del Sacro Cuore, 20123 Rome, Italy; 4 Department of Health Science and Public Health, Faculty of Medicine and Surgery, Catholic University of the Sacred Heart, 20123 Rome, Italy; 5 Clinical Psychology Unit, Ospedale Isola Tiberina–Gemelli Isola, 00186 Rome, Italy; 6 Human Resources and Organization, Fondazione Policlinico Universitario Agostino Gemelli IRCCS, 00168 Rome, Italy; 7 Complex Operational Unit of Clinical and Emergency Psychiatry, Fondazione Policlinico Universitario Agostino Gemelli IRCCS, 00168 Rome, Italy; 8 Department of Neuroscience, Section of Psychiatry, Università Cattolica del Sacro Cuore, 20123 Rome, Italy; 9 Complex Operative Unit of Medical Oncology, Fondazione Policlinico Universitario Agostino Gemelli IRCCS, 00168 Rome, Italy; 10 Scientific Directorate, Fondazione Policlinico Universitario Agostino Gemelli IRCCS, 00168 Rome, Italy; antonio.gasbarrini@policlinicogemelli.it

**Keywords:** clinical psychology unit, integrated care, hospital-wide function, staff wellbeing, personalised medicine, implementation

## Abstract

**Background**: Psychological services within hospitals are essential to delivering integrated, patient-centred care, yet in many health systems they remain fragmented, variably organised, or confined to specific medical specialties. The Clinical Psychology Unit of the Fondazione Policlinico Universitario Agostino Gemelli, Istituto di Ricerca e Cura a Carattere Scientifico (IRCCS), represents one of the few examples of a hospital-wide psychological governance model in Italy, but its organisational structure and longitudinal activity have not previously been systematically described. **Objective**: This study (I) describes the organisational design and operational components of the Gemelli Unit; (II) compares it with international organisational models using a typological framework; and (III) examines its resilience and adaptive capacity during the coronavirus disease 2019 (COVID-19) pandemic. **Methods**: A descriptive–narrative approach was adopted, integrating institutional documentation, routinely collected service data (2019–2024), anonymised case vignettes, and a structured comparison with national and international psychological care structures. The analysis was informed by theoretical models of integrated health-care delivery and by Donabedian’s structure–process–outcome framework. **Results**: Between 2019 and 2024, psychological interventions increased from 28,878 to 47,076 (+63%), with a post-pandemic average of 41,868 annual interventions. In 2024, the Unit supported 2150 patients and 340 healthcare professionals, with psycho-oncology accounting for approximately one-third of all activities. The model integrates clinical activity, staff support, conflict management, research, and training under a centralised governance structure, ensuring hospital-wide coverage and coordinated referral pathways. The comparative analysis identified four international organisational types—department-based, liaison/specialty-based, structured health-system, and academic–clinical hybrid—highlighting the hybrid and transversal nature of the Gemelli Unit and its capacity to maintain and adapt services during the COVID-19 emergency. **Conclusions**: The Gemelli Unit represents a distinctive hospital-wide organisational model that combines centralised governance, transversal deployment, personalised care, and structured support for healthcare professionals. These characteristics position it as a potentially transferable benchmark for health systems seeking to integrate psychological care into core organisational and clinical processes. Future work should prioritise the development of standardised outcome indicators and national frameworks to support the evaluation and harmonisation of hospital-based psychological services.

## 1. Introduction

Clinical Psychology Units (CPUs) are essential components in the integration of mental health services within hospital settings; however, they frequently lack standardised organisational frameworks [1]. This deficiency results in fragmented care, inconsistencies in service delivery, and limited integration within hospital governance and care pathways [2,3].

Despite their recognised importance, hospital-based psychological services often face considerable uncertainty. In many health systems, psychological care is expected to support increasingly complex clinical and organisational demands, yet the structures designed to deliver such care remain fragmented, inconsistently regulated, or restricted to discipline-specific niches. This gap between the growing need for integrated psychosocial support and the limited organisational models available represents a critical tension for contemporary healthcare. Understanding this misalignment is essential for recognising the urgency of developing hospital-wide psychological governance frameworks.

In Italy, the role of psychologists in healthcare was significantly redefined following the establishment of the National Health Service (Servizio Sanitario Nazionale), under Law 833/78 [4,5].

Nonetheless, the absence of national standards for organising psychological services within hospitals has contributed to notable disparities in the availability, scope, and quality of these services [6]. Psychological care is frequently confined to outpatient settings or isolated specialist areas [7], particularly oncology, limiting the systemic integration of psychological perspectives in both patient care and organisational processes [8,9].

Internationally, CPUs exhibit considerable heterogeneity in structure, governance, and operational function [10,11,12]. While certain countries—such as the United Kingdom, Australia, and the United States—have developed more formalised psychology departments with autonomy and defined roles within care pathways, many hospitals still fail to integrate psychological services transversally across departments [13,14,15]. Furthermore, only a few health systems formally recognise psychological services as a hospital-wide organisational responsibility [16,17].

The COVID-19 pandemic further exposed vulnerabilities within mental health services and underscored the need for psychological care to be considered a structural component of healthcare, rather than an emergency-only resource [18,19].

The integration of CPUs can be conceptualised through broader theoretical models of health-service organisation, such as the WHO Integrated Service Delivery framework and Donabedian’s structure–process–outcome model, which emphasise continuity, system governance, and patient-centred design.

Within this framework, the Clinical Psychology Unit of the Fondazione Policlinico Universitario A. Gemelli IRCCS, in Rome, Italy, operates with a dual mandate: (I) clinical and organisational transversality and (II) a hospital-wide function for psychological care. The model integrates psychological support throughout the entire patient journey, including staff wellbeing, conflict management, research, and training.

To date, no reports have provided a systematic organisational description paired with multi-year institutional data (2019–2024) documenting both patient- and staff-focused interventions. This study addresses this gap by (I) outlining the key components of the Gemelli CPU model; (II) comparing it with national and international organisational frameworks; and (III) examining its resilience during the COVID-19 pandemic and its potential for replication in other hospital contexts. Our objective is to contribute to the development of shared organisational standards, advance the integration of psychological care within the core infrastructure of healthcare institutions, and provide insights into internationally transferable models for hospital-based psychological care.

## 2. Materials and Methods

A descriptive–narrative methodology was used to document and analyse the organisational structure, operational principles, and strategic components of the CPU at the Gemelli IRCCS. The methodology approach integrates principles of health-services research and organisational psychology.

### 2.1. Design and Data Sources

The article is based on a qualitative–quantitative organisational analysis that encompasses:

- Internal institutional documentation, including mission statements, organigrams, protocols, and annual activity reports;
- Aggregated service data from the hospital’s digital registry and administrative reporting system (2019–2024), including metrics such as the number of interventions, patient volumes, and staff consultations;
- Retrospective analysis of activity trends and the distribution of interventions across clinical areas;
- Selected case vignettes to illustrate clinical implementation;
- A structured review of national and international CPU models.

In addition, a standardised internal procedure was followed for data selection and extraction. All service data were retrieved from the hospital’s digital administrative registry (TrackCare™), using predefined variables such as date, type of intervention, clinical area, referral source, and professional responsible. Data extraction was performed by two trained administrative officers and cross-checked by a senior psychologist to ensure accuracy, completeness, and consistency. Only aggregated and fully anonymized data were included.

To enhance clarity and prevent methodological overload, descriptive procedures not essential to transparency or reproducibility were summarised. This streamlined presentation follows recommendations for narrative organisational research and aims to facilitate reader comprehension.

### 2.2. Comparative Analysis Criteria

A comparative matrix was developed to analyse CPUs across countries, using indicators such as:

- Level integration within hospital governance (vertical versus transversal positioning);
- Scope of activity (clinical care, training, research, staff support);
- Governance and coordination mechanisms;
- The availability of a centralised referral system;
- The involvement in emergency response initiatives, such as during the COVID-19 pandemic.

Countries and models were selected based on documentation availability, presence of formal psychology services, relevance to Western health-system contexts, and diversity of organisational models (centralised, department-based, liaison-oriented). This approach aligns with comparative health-system analysis frameworks and supports the transferability evaluation of the Gemelli model.

### 2.3. Ethical Considerations

Only anonymized, organisational data were analysed. The study did not involve any intervention or access to identifiable patient information. The Institutional Review Board of the Fondazione Policlinico Universitario A. Gemelli IRCCS confirmed that the study did not constitute human-subjects research and did not require ethics approval. Institutional authorisation for the use of aggregated data and de-identified vignettes was obtained.

### 2.4. Reporting Standards

The structure and content of this report follow narrative reporting standards appropriate for qualitative and organisational health services research. Specifically, the guidelines set forth by Greenhalgh et al. [20] were followed, which emphasise the importance of reflexivity, contextualization, and transferability when describing complex service models. Methodological guidance from Pope and Mays [21] was also applied to support the systematic presentation of institutional case studies. This approach ensured transparent reporting of data sources, a clear rationale for the inclusion of illustrative clinical vignettes, and an explicit justification for the development of the comparative matrix. The narrative is organised to enable readers to understand the organisational decisions taken, assess their potential for replication, and situate them within the broader discourse on hospital-based psychological care.

Additional reporting considerations were informed by the Consolidated Criteria for Reporting Qualitative Research (COREQ) and the Standards for Reporting Qualitative Research (SRQR), ensuring transparency regarding context, researcher role, and institutional positioning.

### 2.5. Professionals Involved

The CPU is staffed by 35 licenced clinical psychologists (24 full-time equivalents), distributed across various clinical areas including oncology, neurology, paediatrics, emergency medicine, transplant medicine, and chronic disease management. The team comprises senior psychologists, early-career professionals, and specialists in neuropsychology and psychotherapy. Each psychologist is responsible for a specific clinical area and contributes to staff support, clinical activity, and training according to their expertise and workload.

## 3. Results

### 3.1. Overview of the Gemelli CPU Model

To ensure analytical coherence and alignment with the study objectives, Section 3 is organised according to the three predefined aims: (I) a description of the organisational model; (II) a longitudinal analysis of activity data; and (III) a comparative positioning within national and international frameworks. This structure mirrors the methodological approach and clarifies the logic guiding the presentation of findings.

The CPU of the Fondazione Policlinico Universitario A. Gemelli IRCCS operates a hospital-wide service grounded in principles of clinical and organisational transversality, integrating psychological support across all departments. Rather than functioning as an isolated department, the CPU provides services throughout the hospitals, ensuring that psychological support is embedded within clinical pathways from admission to discharge and, where appropriate, into post-discharge follow-up.

As shown in Table 1, the model is built upon four strategic pillars, Clinical Care, Research, Training, and Conflict Management, each implemented across a broad range of medical and organisational contexts. This transversal structure supports a high degree of personalization, enabling interventions to be adapted to diverse patient trajectories, clinical conditions, and psychosocial contexts.

### 3.2. Operational Structure and Coordination

The CPU is directed by a Clinical Director and organised around area coordinators who oversee and supervise interventions. Referrals are managed through a centralised internal digital platform that enables the assignment of psychologists according to their specific areas of expertise, availability, and the need to ensure continuity of care. This allocation system ensures that patients and staff receive interventions tailored to their individual needs, clinical histories, and relational contexts. It functions as a personalised matching mechanism that optimises resources use while maintaining therapeutic continuity, relational familiarity, and adaptive responsiveness during emergencies [22]. The essential structural and procedural components of the CPU model are outlined in Table 2.

Furthermore, the allocation system is monitored by area coordinators, who conduct quality checks on referral triage, urgency classification, and assignment accuracy. This oversight ensures consistency across units and minimises variability in access, thereby supporting equitable and efficient service delivery.

### 3.3. Summary of Activities and Reach

From 2019 to 2024, the CPU demonstrated a sustained and quantifiable expansion of its activities across the hospital system. To enhance readability and support rapid comprehension of activity trends, a concise overview of the main longitudinal patterns is presented prior to the detailed numerical tables. Overall activity increased steadily over the six-year period, reaching a peak of 47,076 interventions in 2024, representing a 63% rise compared with 2019. The post-pandemic period showed an average of 41,868 annual interventions, reflecting sustained structural growth. In 2024, the CPU supported 2150 patients and 340 healthcare professionals, with psycho-oncology accounting for approximately one-third of all activities (see Table 3). Despite a temporary reduction in service delivery during the COVID-19 pandemic in 2020—when interventions dropped to 24,708, a 14.5% decline from 2019- subsequent years exhibited substantial recovery and structural expansion. The post-pandemic average of 41,868 annual interventions exceeded the pre-pandemic biennium average of 26,793, corresponding to a 56% increase. This trend underscores not only the resilience of the Gemelli CPU model but also its capacity to deliver personalised psychological interventions across diverse systemic conditions, thereby supporting both scalability and patient-centred care. The compound annual growth rate (CAGR) from 2019 to 2024 was 10.3%.

In 2024, the CPU assisted 2150 patients, facilitated 16,650 initial consultations, and delivered a total of 47,076 interventions, corresponding to an average of 4280 sessions per month. Each patient received an average of 7.7 clinical consultations, highlighting the Unit’s commitment to maintaining continuity of care beyond the initial assessment. Concurrently, 340 healthcare professionals received targeted support, reflecting the essential role of CPU in promoting staff wellbeing and enhancing organisational functioning. Staff interventions were tailored to department-specific stressors, professional roles, and individual needs, aligning with personalised preventive mental health strategies.

CPU services spanned more than 30 clinical specialties, including psycho-oncology, neuropsychology, maternal and child health, emergency and critical care, psychiatry, transplant medicine, and chronic disease management. Psycho-oncology represented the largest activity area, with gynaecological oncology (35%) and breast cancer (30%) collectively accounting for nearly two-thirds of the interventions in this category (see Table 4). This distribution reflects both the epidemiological burden of oncological diseases and the CPU’s ability to tailor interventions to patient-specific care trajectories, such as anticipatory grief in oncofertility, resilience-building during long-term survivorship, and different support across oncological pathways.

Overall, psycho-oncology accounted for approximately one-third of all CPU activities in 2024, with a predominant focus on gynaecological and breast cancers. In total, 2150 patients and 340 healthcare professionals received support, amounting to 2490 individuals assisted. This corresponds to a patient-to-staff ratio of 6.3:1, with staff support representing nearly 15% of all interventions. This dual focus—addressing both patients’ needs and structured psychological support to healthcare workers—distinguishes the Gemelli CPU, a feature seldom documented in comparable international frameworks.

The cumulative findings highlight the operational effectiveness, cross-sectoral integration, and clinical significance of the Gemelli CPU. The documented growth in service volume, diversification of clinical targets, and systematic engagement with both patients and healthcare staff position this model as a scalable and replicable example of hospital-based psychological care. Importantly, the combination of quantitative expansion and personalised intervention strategies illustrates how the CPU translates the principles of personalised medicine into everyday clinical practice within hospital systems.

To further illustrate the practical implementation of the CPU model, two clinical vignettes are provided (refer to Box 1).

Box 1 **Case vignette illustrating the implementation of the Gemelli CPU model**

**Case 1—Post-Traumatic Brain Injury and Rehabilitation.** A 28-year-old male was admitted to intensive care after a motorcycle accident resulting in multiple thoracic and neurological injuries. The CPU intervened within 48 h, focusing on trauma processing, orientation, and family psychoeducation. During neurorehabilitation, psychological assessments (HADS, MoCA, Mental Deterioration Battery) revealed emotional distress and cognitive impairments. Tailored trauma-focused therapy and cognitive support were provided. Interdisciplinary coordination facilitated recovery and helped **prevent the onset of post-traumatic stress disorder (PTSD).**

**Case 2—Oncofertility and Psychological Mourning.** A 36-year-old woman was referred to the oncofertility service before undergoing pelvic surgery for cervical cancer. Although her initial HADS scores fell within non-pathological ranges, anticipatory grief associated with potential fertility loss was evident. CPU interventions addressed this mourning process and ensured continuity of care across pre- and post-operative stages. Following treatment, her distress intensified, prompting psychiatric consultation and pharmacological support. Ongoing psycho-oncological engagement promoted resilience, mitigated the risk of trauma-related psychopathology, and supported recovery.

### 3.4. Comparative Perspective

To situate the Gemelli CPU model within a broader international context, a structured comparative analysis was conducted with similar models operating in Italy, the United Kingdom, the United States, Australia, and selected European, Asian, and African health systems. The sources consulted for this analysis included institutional documents, reports from psychological associations, and relevant scientific literature [23,24,25,26,27,28,29,30,31,32,33,34,35].

To enhance interpretability, international services were grouped into four broad organisational typologies, reflecting recurrent structural patterns across health systems (Table 5). For example, liaison psychiatry practices in France [27], specialist-driven units such as the FIT-hospital model in Germany [28], and mental health integration initiatives in Spain, including the National Mental Health Strategy [29], provide valuable clinical coverage but seldom achieve full hospital-wide transversality. Similarly, while models in the United Kingdom and the United States demonstrate stronger outcomes monitoring and closer integration between academic and clinical frameworks, the implementation of staff-support programmes and overarching hospital governance remains inconsistent [36,37,38,39]. Extra-European examples, such as those from Singapore and South Africa, illustrate innovative approaches but also highlight substantial variability in structural integration [30,31,32,33,34,35].

Within this typological landscape, the Gemelli CPU aligns most closely with a hybrid model that combines hospital-wide governance, transversal deployment, academic integration, and structured staff-support programmes—an organisational configuration rarely documented in the literature. Its centralised leadership, digital referral system, cross-sectoral continuity, and demonstrated resilience during the COVID-19 emergency clearly differentiate it from both department-based and liaison-based frameworks.

Furthermore, the explicit personalization of psychological interventions—linking patient trajectories, family systems, and staff-specific stressors—positions the Gemelli CPU as a transferable benchmark for hospital-based psychological care, particularly for health systems seeking to embed personalised medicine principles within their organisational design.

Taken together, these findings outline a coherent organisational, operational, and activity-based profile of the Gemelli CPU, providing the empirical foundation needed to interpret the model’s functioning in relation to the study objectives. This structured set of results also establishes the analytical basis for the subsequent Discussion, where the organisational implications, theoretical connections, and comparative significance of the data are examined.

## 4. Discussion

This Discussion revisits the study’s three objectives—describing the organisational model of the Gemelli CPU, analysing longitudinal activity trends, and positioning the Unit within international frameworks—in order to contextualise the findings and clarify their contribution to hospital-based psychological care.

The CPU model implemented at the Fondazione Policlinico Universitario A. Gemelli IRCCS constitutes a sophisticated organisational framework designed to address longstanding challenges in hospital-based psychological care. Unlike many existing services, which remain confined to specific departments or diagnostic categories [40,41], the Gemelli CPU is characterised by centralised governance and cross-departmental integration. These features enable the systematic incorporation of psychological support throughout the entire care pathway, promoting continuity, personalization, and adaptability. Interpreting the findings through established frameworks of integrated care—such as the WHO Integrated Service Delivery model and Donabedian’s structure–process–outcome framework- further strengthens the conceptual grounding of this model, both of which emphasise system-level organisation and transversal governance as prerequisites for effective service delivery.

From a systems perspective, this model aligns with contemporary demands for integrated, patient-centred care [42,43]. By harmonising clinical care, staff training, research, and conflict management, the CPU simultaneously addresses the wellbeing of individuals and the broader functioning of the organisation. Notably, approximately 15% of its activities are dedicated to staff support—a dimension rarely documented in comparable frameworks. This dual focus on both patients and healthcare professionals represents a meaningful innovation, underscoring the strategic importance of psychological services as a core organisational resource. The personalised tailoring of staff interventions—differentiated according to professional role, department stressors, and crisis contexts—demonstrates how the CPU extends the principles of personalised medicine beyond patients to include the workforce.

The operational flexibility demonstrated during the COVID-19 pandemic further validated the resilience of this organisational model. Rapid transition to telepsychology, targeted support during staff crises, and the preservation of service continuity exemplify the CPU’s ability to adapt under systemic strain [44,45]. This adaptability underscores the necessity of structural integrated psychological services for maintaining hospital-wide functioning during emergencies [46,47]. Importantly, the capacity to individualise interventions—from trauma-focused support for patients with acute injuries to anticipatory grief counselling in oncology—illustrates the model’s ability to provide person-tailored care in both crisis and routine conditions.

Taken together, these operational and clinical patterns offer a coherent empirical basis for understanding how the CPU functions as an integrated hospital-wide system, bridging the descriptive results to the broader organisational and theoretical considerations explored in this Discussed.

To align the interpretation more explicitly with the study objectives, the Discussion is structured around the three predefined aims. First, the description of the CPU model confirms that centralised governance and transversal deployment constitute structural mechanisms that support both scalability and continuity of care. Second, the comparative analysis, supported by the typological framework presented in Table 5, clarifies how international models cluster into department-based, liaison-based, structured health-system, and academic–clinical hybrid configurations. Within this landscape, the Gemelli CPU occupies a hybrid organisational position that combines high-level governance, academic integration, and systematic staff-support programmes—an uncommon configuration that distinguishes it from most existing services.

Furthermore, interpreting the comparative findings through the lens of integrated-care theory strengthens the argument for the model’s transferability. Systems with weak hospital-wide governance or discipline-specific deployment (e.g., liaison or specialist-based structures) inherently limit continuity, while structured systems (e.g., NHS, Australia) often enhance monitoring but lack consistent staff-support mechanisms. These contrasts help clarify why the Gemelli CPU may serve as a unique organisational benchmark.

Internationally, few hospital-based psychological services successfully combine centralised leadership, cross-sectoral outreach, and structured staff wellbeing programmes [48]. Most existing systems remain fragmented or specialty-specific, such as liaison psychiatry models in France, specialist-led frameworks in Germany, or partially integrated services in Spain [27,28,29]. In the UK and the US, although outcome monitoring is generally more robust, governance and staff-support are structures remain inconsistent [36,39]. Within this context, the Gemelli CPU presents a replicable organisational model capable of meeting both clinical and institutional demands. A distinguishing feature is the systematic personalization of interventions, bridging clinical, psychosocial, and organisational dimensions. This illustrates how the principles of personalised medicine principles can be embedded in hospital governance and operational practice.

Certain limitations should be acknowledged. The absence of standardised outcome indicators and longitudinal measures limits the empirical evaluation of the model’s effectiveness. Additionally, the lack of a national regulatory framework for CPUs in Italy hinders the harmonisation and scalability of such models. Future initiatives should prioritise the development of shared benchmarks and outcome metrics specifically tailored to hospital-based psychological care [49], including personalised outcome measures such as patient-reported outcomes (PROs), staff wellbeing indicators, and longitudinal metrics capturing individual trajectories of change.

Future work should therefore focus on the development of measurable, theoretically grounded indicators capable of assessing organisational effectiveness (structure), quality of clinical work (process), and patient and staff outcomes, in accordance with Donabedian’s tripartite model.

In summary, the Gemelli CPU exemplifies the potential evolution of psychological services from ancillary support to an essential structural components within contemporary healthcare systems. By integrating clinical and organisational functions under a centralised framework, the CPU establishes a paradigm in which psychosocial care becomes fundamental for both patients and healthcare professionals, thereby contributing to the transformation of hospital systems. Through its commitment to personalization—tailoring interventions to heterogeneous patient populations, clinical pathways, and staff roles—the CPU demonstrates how personalised medicine can be applied to psychological care at scale.

Overall, these elements illustrate how the Gemelli CPU operates not only as a clinical service but as a strategic organisational mechanism, setting the stage for the concluding reflections on its broader implications and future directions.

## 5. Conclusions

This study set out to describe the organisational design of the Gemelli CPU, analyse its multi-year activity trends, and situate the model within international frameworks. The findings demonstrate that the Gemelli CPU illustrates the potential for hospital-based psychological services to become an integral component of contemporary healthcare systems. By integrating clinical care, staff wellbeing, training, and research within a unified governance framework, the model effectively addresses the needs of both patients and organisations. Its adaptability during the COVID-19 pandemic, together with its dual focus on patients and healthcare professionals, underscores its scalability and replicability.

Consistent with the study objectives, this organisational analysis provides three primary contributions: (I) a detailed description of a hospital-wide psychological governance model; (II) a typological comparison that elucidates how international services cluster into distinct organisational configurations; and (III) an examination of system resilience that highlights the structural features enabling continuity, personalisation, and crisis responsiveness. These elements collectively position the Gemelli CPU as a hybrid model that integrates governance, clinical activity, and staff support in ways rarely documented in the literature.

Future developments should prioritise the establishment of standardised outcome metrics and national frameworks to support broader implementation and reinforce the role of psychological care as a fundamental component of healthcare systems. In particular, the creation of theory-informed indicators—drawing on integrated-care models and Donabedian’s structure–process–outcome framework—will be essential for evaluating organisational effectiveness, continuity of care, and the trajectories of both patient and staff over time.

Furthermore, the typological perspective adopted in this study offers a transferable analytical tool for other health systems seeking to assess or redesign their psychological care structures. By categorising international models into four organisational types, the analysis clarifies where fragmentation persists, where governance is structurally embedded, and where research and staff-support remain underdeveloped.

In conclusion, this work provides a theoretically grounded and empirically supported framework for understanding how hospital-wide psychological governance can be effectively implemented, demonstrating how organisational design can translate the principles of personalised medicine into diversified, scalable, and system-level psychological care.

## Figures and Tables

**Table 1:** Strategic Functions of the Gemelli Clinical Psychology Unit (CPU).

Function	Operational Description	Examples
**Clinical Care**	Delivery of psychological support across all hospital units, from acute care to follow-up	Individual therapy, crisis intervention, psychodiagnostics
**Research**	Active participation in clinical studies, adherence protocols, and quality-of-life research	Multicentre projects, treatment outcome monitoring
**Training**	Continuous education of medical staff in psychological and relational competencies	Workshops, conflict resolution training, and reflective supervision
**Conflict Management**	Structured response to interpersonal and institutional tensions	Peer mediation, burnout prevention, and post-incident debriefing

Overview of the four core functions that structure the CPU model, illustrating their operational scope and concrete examples of implementation across the hospital setting.

**Table 2:** Operational Framework of the Gemelli Clinical Psychology Unit.

Component	Description
**Referral System**	A centralised digital platform integrated into the hospital intranet allows medical teams to request psychological interventions directly. This system ensures traceability, prioritisation, and timely response.
**Professional Allocation**	Psychologists are assigned based on specific expertise in clinical areas (e.g., oncology, neurology, paediatrics). Allocation considers continuity of care, relational familiarity, and workload distribution.
**Target Populations**	The CPU provides support to inpatients, outpatients, caregivers, and healthcare staff. Particular attention is given to vulnerable populations and high-intensity care settings.
**Assessment Tools**	A broad set of validated tools is used, including the Hospital Anxiety and Depression Scale (HADS) for anxiety/depression screening, the Montreal Cognitive Assessment (MoCA) for cognitive screening, the Mental Deterioration Battery (MBD) and the Wechsler Adult Intelligence Scale (WAIS) for neuropsychological assessment, the Vineland Adaptive Behavior Scales for adaptive functioning, and structured clinical interviews.
**Intervention Formats**	Interventions range from brief to long-term formats and include individual therapy, family counselling, group interventions, telepsychology, and crisis-focused sessions in acute care units.

Detailed overview of the structural and functional components that enable the implementation of the CPU model across the hospital system.

**Table 3:** Annual clinical activities of the Gemelli Clinical Psychology Unit (2019–2024).

Year	Total Interventions	Δ vs. Previous Year	Notes
2019	28,878	–	Baseline (pre-COVID)
2020	24,708	–14.5%	COVID-19 impact
2021	35,345	+43.1%	Post-pandemic recovery
2022	36,471	+3.2%	Consolidation
2023	45,581	+24.9%	Expansion
2024	47,076	+3.3%	Record activity level

Data refer to the period October 2019–November 2024. Overall increase 2019–2024 = +63%; compound annual growth rate (CAGR) = 10.3%.

**Table 4:** Distribution of psycho-oncology interventions by specialty (2024).

Specialty Area	% of Total Psycho-Oncology Interventions
Gynaecological oncology	35%
Breast cancer (Senology)	30%
Pancreas	10%
Gastroenterology	9%
Central nervous system	7%
Haematology–Oncology	9%

Note. Percentages refer to the distribution of psycho-oncology activities within the CPU in 2024.

**Table 5:** Typology of international Clinical Psychology Unit models and position of the Gemelli.

Model Type	Definition	Countries/Examples	Key Features	Contrast with Gemelli CPU
1. Department-Based Model	Psychology operates as a stand-alone department with limited cross-unit involvement	Italy (most CPUs), Spain, Germany	- Fragmented activities - Limited governance role - Specialty-focused	Gemelli is hospital-wide, centralised, and transversal
2. Liaison/Specialty Model	Psychologists embedded in specific medical units without system-wide mandate	France (liaison psychiatry), many EU hospitals	- High expertise in one area - No central referral system - Weak organisational integration	Gemelli integrates all specialties under one governance
3. Structured Health-System Model	Formalised psychology departments integrated into care pathways	United Kingdom National Health Service (NHS) and Australian health-system models	- Strong protocols and monitoring - Better research culture - Variable staff support	Gemelli has stronger staff programmes and broader hospital remit
4. Academic–Clinical Hybrid Model	Integrated with universities, strong research–practice link	USA, Singapore	- Outcome monitoring - Research embedded - Variable governance	Gemelli combines governance + research + staff care, rarely unified elsewhere
GEMELLI CPU (Benchmark)	Centralised, hospital-wide, transversal psychological governance	Italy (Policlinico Gemelli IRCCS)	- Digital referral system - Staff support programme - Emergency resilience (COVID-19) - Structured governance	Unique combination of hospital-wide mandate + staff programmes + transversal integration

Note: This typological table highlights the conceptual differences among international models and illustrates how the Gemelli CPU represents a hybrid and structurally distinctive framework.

## Data Availability

The raw data supporting the conclusions of this article will be made available by the authors on request.
